# Managing a Complex Pediatric Airway Affected by Venolymphatic Malformation of the Face: A Case Report

**DOI:** 10.7759/cureus.79096

**Published:** 2025-02-16

**Authors:** Sonam Patel, Vijeta Bajpai, Priyanka Dwivedi, Ankita Kabi, Ganesh R Nimje

**Affiliations:** 1 Department of Anesthesiology, Pain Medicine and Critical Care, All India Institute of Medical Sciences, Gorakhpur, IND

**Keywords:** difficult intubation, difficult mask ventilation, pediatric airway management, supraglottic airway device, venolymphatic malformation

## Abstract

We present a case of a two-year-old male child with a progressive venolymphatic malformation on the right cheek, resulting in facial asymmetry and mass effect, scheduled for mass excision under general anesthesia. The preoperative assessment indicated a potential for difficult mask ventilation and intubation due to the deviation of the mouth. Airway management strategies included two-hand mask techniques, appropriately sized masks, supraglottic airway device (SGAD) insertion during spontaneous respiration, video laryngoscopy, and awake fiberoptic intubation. During induction, an SGAD (i-gel no. 2) was successfully placed under spontaneous breathing, and after checking the adequacy of ventilation, endotracheal intubation with muscle relaxation was done. The surgery proceeded without complications, and the patient was extubated in the postoperative period. This case emphasizes the importance of successfully managing airway challenges through individualized planning in complex pediatric airways, especially in patients with facial malformations.

## Introduction

Venolymphatic malformations (VLMs) are congenital slow-flow vascular malformations (VMs) that result from abnormal connections between blood vessels, which can occur in various parts of the body. Notably, approximately 60% of these malformations are located in the maxillofacial region [1]. Although benign, VMs in the head and neck can progressively enlarge over time, leading to tissue destruction and functional challenges. These may include nasal obstruction, speech difficulty, dental complications, and significant cosmetic disfigurement. Huge VMs, particularly on the lips, cheeks, and tongue, make airway management very challenging, as they cause anatomical changes leading to poor face mask seal and deviation of oropharyngolaryngeal axis, which further poses difficulty in mask ventilation and intubation, especially in a pediatric patient [2,3]. The treatment of these VMs varies depending on the blood flow, utilizing sclerotherapy for low-flow lesions, embolization for high-flow lesions, and surgical corrections in complex cases [4]. This case report highlights the unique challenges of airway management in a child presenting with a large facial swelling, diagnosed as a VLM on the right cheek.

## Case presentation

**Figure 1:**
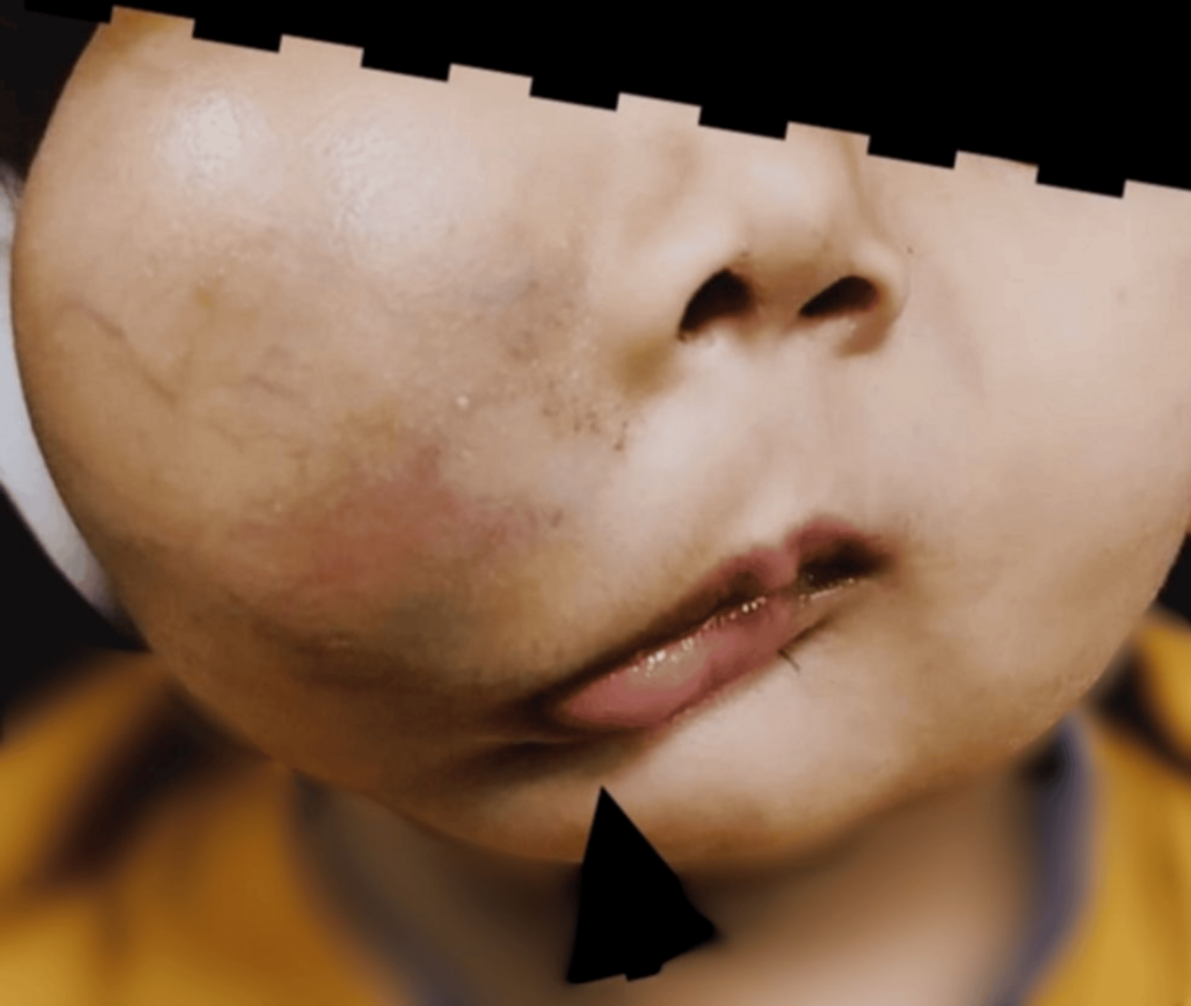
Anticipated difficult mask ventilation due to large swelling Large swelling on the right side of the cheek extending superiorly up to the medial canthus of the eye with a deviation of the angle of the mouth (black arrowhead) is noticed

**Figure 2:**
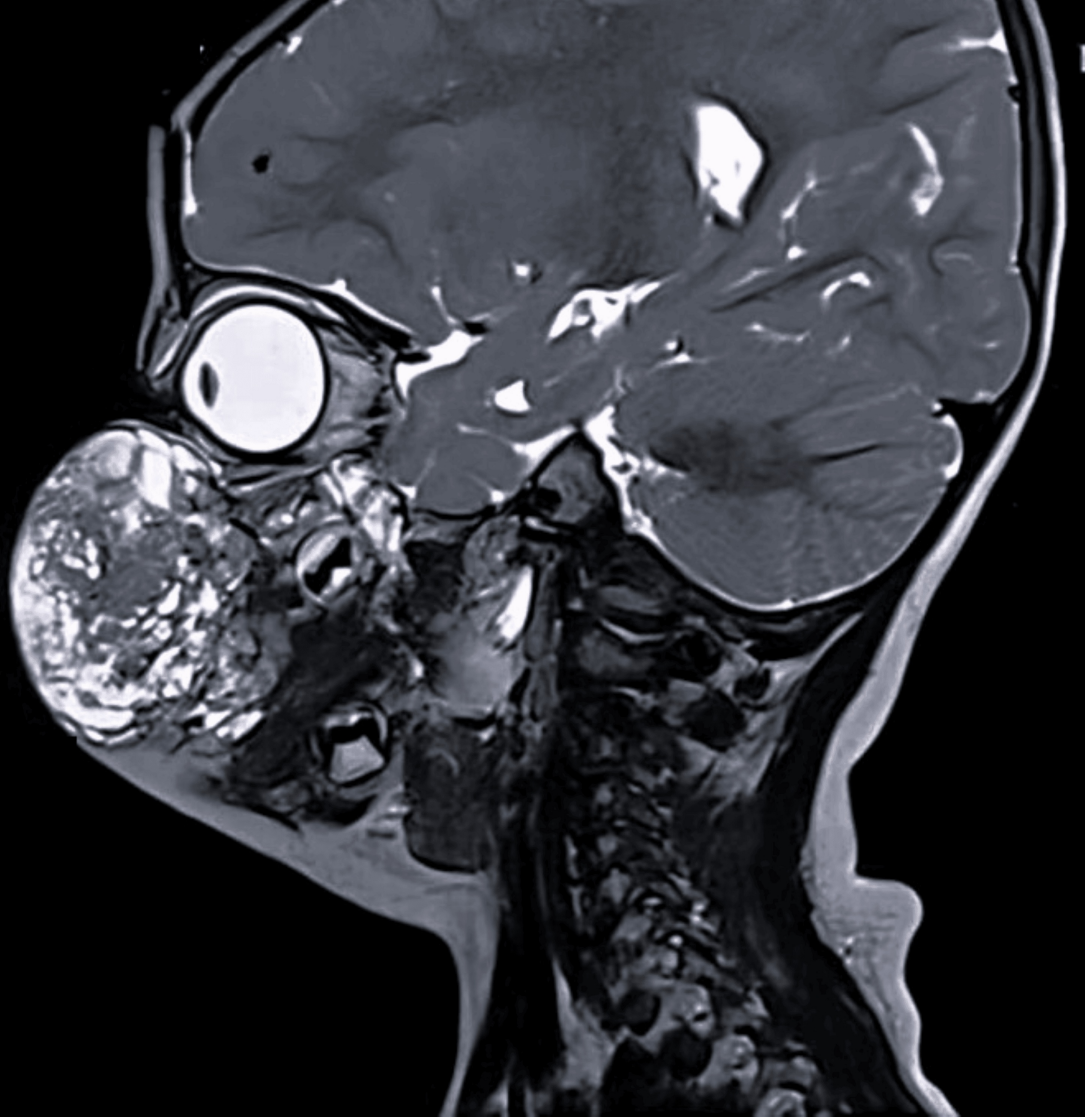
MRI scan showing a lesion of size 3.2 × 5.6 × 5.2 cm involving skin and subcutaneous tissue of the right side of the cheek extending superiorly on the posterior aspect of zygomatic bones, retromaxillary space, up to the level of sphenopalatine foramina and medially up to the medial canthus of the right eye MRI: magnetic resonance imaging

A two-year-old male child, weighing 10.2 kg, presented with swelling over the right cheek since birth, which had gradually increased in size. There was no history of difficulty swallowing or breathing in any position (supine or lateral). Furthermore, there was no history of snoring during sleep; however, it caused significant facial deformity (Figure 1). The preanesthetic evaluation revealed a normal birth and developmental history. Systemic examination and blood tests were age-appropriate. A magnetic resonance imaging scan revealed a lesion measuring 3.2 x 5.6 x 5.2 cm involving the right cheek, extending superiorly on the posterior aspect of the zygomatic bones and the retromaxillary space up to the level of the sphenopalatine foramina and medially to the medial canthus of the right eye (Figure 2). No retro-/intraorbital or intracranial extension was observed. The Doppler study suggested VLM. The child was scheduled for mass excision under general anesthesia with endotracheal intubation.

During the airway examination, the mouth opening was two fingers wide. However, the Mallampati grading could not be assessed due to a deviation of the mouth to the left side. Difficulties with mask ventilation and intubation were anticipated; therefore, we planned to avoid inducing apnea and to secure the airway by inserting a supraglottic airway device (SGAD) while maintaining spontaneous respiration. Once the adequacy of ventilation with the SGAD is ensured, either conventional or video laryngoscopy followed by endotracheal intubation will be performed (plan A). Alternatively, we have planned for airway management with awake flexible fiberoptic intubation if there is any difficulty in mask ventilation or inserting the SGAD at any step (plan B). Additionally, the case was discussed with the Ear, Nose, and Throat (ENT) Surgeon for backup in case of any airway emergency (plan C).

**Figure 3:**
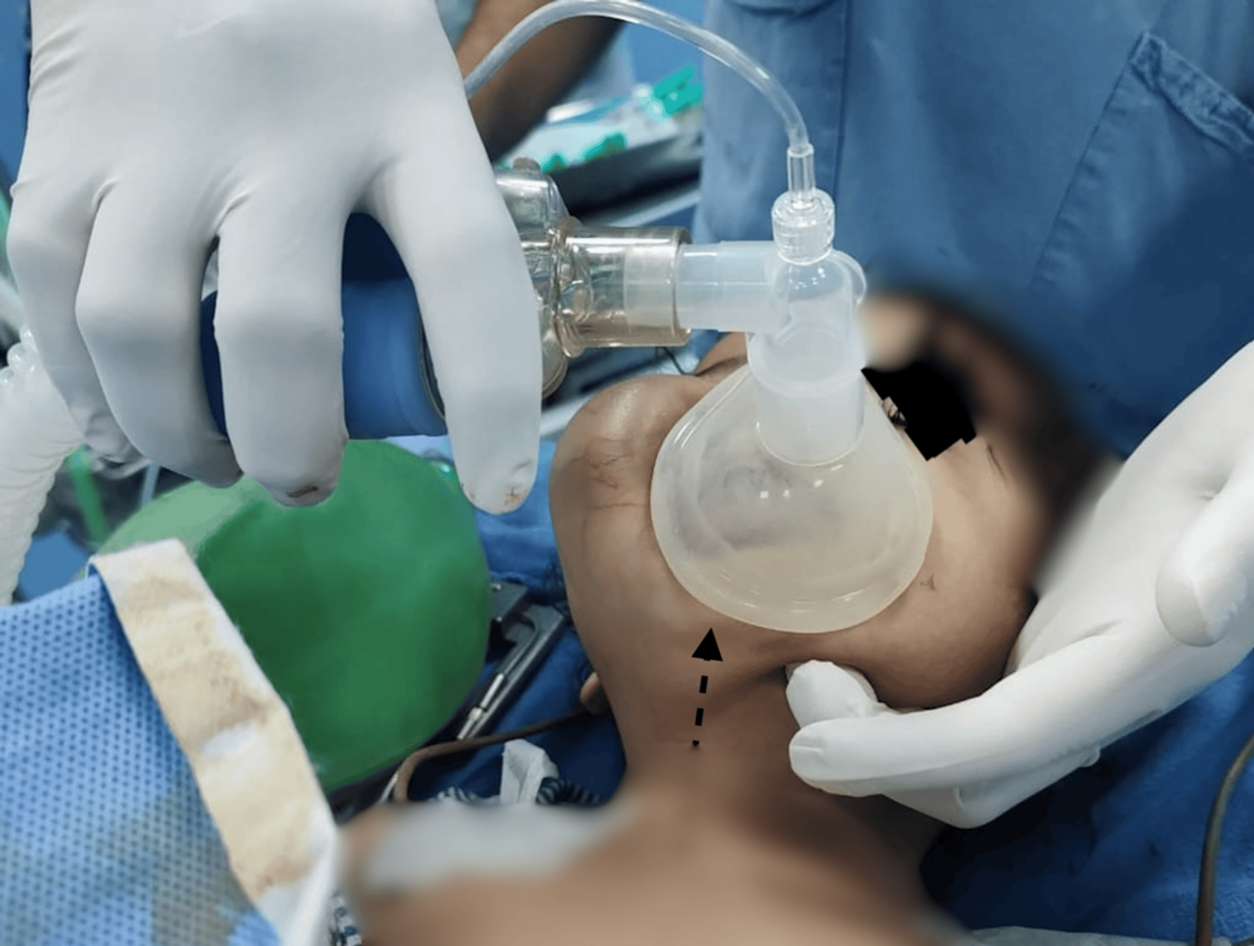
Difficult mask ventilation due to swelling Poor-fitting of the face mask due to facial disfigurement (black arrow)

On the day of surgery, after confirming nil per oral status and obtaining consent from the parents, the patient was nebulized with 1 mL of lignocaine 2%, followed by premedication with ketamine (4 mg/kg intramuscular) and glycopyrrolate (4 mg/kg IV) in the preoperative room. In the operation theater, a difficult airway cart with appropriately sized masks, a pediatric fiberoptic bronchoscope, and backup support from the ENT team for emergency airway management was prepared. For preoxygenation, a pediatric size 2 silicone mask was used; however, the seal was inadequate. Consequently, a larger mask with a two-hand technique was applied, but there was still a constant leak due to the poor fit of the mask (Figure 3). Considering the potential for leakage of the inhalational agent, slow intravenous (IV) induction with propofol at 2 mg /kg instead of inhalation induction was used. During spontaneous respiration, an i-gel SGAD (no. 2) was inserted to secure the airway. After confirming the adequacy of ventilation, fentanyl 10 mcg and atracurium 5 mg were administered, and endotracheal intubation was successfully performed using an endotracheal tube (ETT) size 4 following the removal of the i-gel. Correct ETT placement was confirmed through bilateral auscultation and end-tidal CO_2_ waveforms. Anesthesia was maintained with sevoflurane at 1-1.2 minimum alveolar concentration in an air-oxygen mixture (1:1), with end-tidal CO_2_ levels maintained between 35 and 45 mm Hg. Fentanyl 10 mcg was repeated as and when required. The intraoperative period was uneventful, and the surgery lasted for one hour. After completion of the procedure, the child was extubated when fully awake and with adequate muscle strength. His postoperative period was unremarkable, and the child was discharged home on the fifth postoperative day.

## Discussion

Pediatric patients present unique anatomical and physiological considerations in airway management, which impose significant limitations on safe apnea time before the onset of hypoxemia and subsequent bradycardia [5]. Also, airway examination is challenging due to poor cooperation and the unreliability of the Mallampati scoring for predicting difficult airways in children [6]. Huge facial swellings (intraoral or extraoral), craniofacial syndromes, or facial trauma impose added challenges in pediatric patients, as effective face mask ventilation is crucial to avoid hypoxemia and adverse events, particularly when tracheal intubation is also challenging. General anesthesia with an inhaled agent such as sevoflurane or an IV agent (such as propofol, ketamine, or dexmedetomidine) while maintaining spontaneous ventilation is the technique of choice in such cases [7]. In this case, difficulty with bag-and-mask ventilation was anticipated due to the large swelling of the face. Therefore, a difficult airway cart with appropriately sized masks, small gauge airways, and a pediatric flexible fiberoptic bronchoscope was prepared. The child was nebulized with lignocaine in the preoperative area as part of the preparation for fiberoptic intubation. The airway management plan was discussed with the ENT team and kept on standby in the operating room. For preoxygenation, mask ventilation with a larger mask and a two-hand technique was used to achieve a proper seal; however, it was unsuccessful. Therefore, a decision was taken not to abolish airway reflexes and induce apnea. Also, since the mask seal was inappropriate, inhalational induction was bypassed, and i-gel SGAD was used as a rescue device before giving muscle relaxant. After confirming ventilation adequacy with waveform capnography, further muscle relaxant was given, and endotracheal intubation was done successfully.

The use of SGAD for airway management is a useful option for children if difficulty in the visualization of the vocal cords is anticipated. It also offers fewer hemodynamic effects and lesser chances of airway-related complications, which makes it a safe option for short-duration surgeries. In our case, since the vascular anomaly was not extending into the oral cavity, we inserted i-gel SGAD to secure the airway. Furthermore, as the surgical site was in close proximity to the patient’s airway, there were chances of displacement of SGAD during the surgical procedure; thus, it was not considered a definitive airway, and the decision was taken to do laryngoscopy and intubation. If the check laryngoscopy proved difficult or the view of the larynx was poor, we would have awakened the child and proceeded with awake fiberoptic intubation. Additionally, in the event of any airway emergency, the ENT team was prepared for an emergent tracheostomy.

A recent network meta-analysis [8] evaluated the effectiveness of 13 different intubation devices in pediatric patients. The findings indicate that no laryngoscope currently outperforms the Macintosh laryngoscope in terms of tracheal intubation failure rate and glottic visualization. However, certain video laryngoscopes were associated with significantly shorter intubation times. In our patient, endotracheal intubation was successfully performed on the first attempt using gentle laryngoscopy with a Macintosh blade.

Flexible fiberoptic intubation is an important option for patients with anticipated airway difficulty. However, it is not always the first choice in children due to their lack of cooperation, anatomical factors like a proportionally larger tongue, and increased oxygen consumption, which makes brief apneic periods during the procedure challenging to tolerate. Also, the smaller pediatric airways make the manipulation of the fiberoptic more difficult [9,10]. In our case, we did not consider it the preferred option for managing the airway.

Literature reports complications after procedures for venous malformations, particularly airway edema that can lead to obstruction and extubation failure [11]. A meta-analysis conducted by De Maria et al. [12] reported that temporary local swelling rates were high after sclerotherapy injection due to interstitial edema. In our case, the extubation plan was discussed with the operating surgeon at the completion of surgery. Since the surgical procedure was entirely extraoral and did not involve the use of a sclerosing agent, there was minimal chance of airway threat due to surrounding tissue edema. Therefore, the decision was made to extubate the child.

Unlike the American Society of Anesthesiologists' adult difficult airway algorithm, airway management in infants and children with difficult airways lacks a standardized approach. Therefore, individualized anesthesia planning for the safe and effective management of airways is crucial in suspected difficult airways, especially in children with craniofacial deformities or disfigurements.

## Conclusions

The airway management of children with facial disfigurement imposes great challenges owing to the difficulties in mask ventilation and potentially difficult intubation. Preserving spontaneous breathing is one of the safest strategies when patients are at risk of difficult ventilation. This case highlights the importance of preoperative planning, backup strategies, and the adaptability of airway techniques in ensuring patient safety. In this case, the use of appropriately sized masks, the two-handed mask ventilation technique, and SGAD followed by endotracheal intubation highlights the need to tailor airway management techniques to the anticipated difficulty and the provider’s skill and experience, ensuring optimal oxygen delivery and minimal physiological disruption. If one approach fails, an alternative strategy should be promptly implemented to reduce the number of attempts and quickly restore oxygenation and ventilation.
